# The Substantial Role of Sleep, Stress, and Physical Activity in Persistent High Levels of Fatigue in Patients With Inflammatory Bowel Disease: A Longitudinal Trajectory Study

**DOI:** 10.1093/ecco-jcc/jjae163

**Published:** 2024-10-26

**Authors:** Quirine M Bredero, Joke Fleer, Denise M Blom, Arno R Bourgonje, Gerard Dijkstra, Maya J Schroevers

**Affiliations:** Department of Health Sciences, Unit Health Psychology, University Medical Centre Groningen, University of Groningen, Groningen, The Netherlands; Department of Health Sciences, Unit Health Psychology, University Medical Centre Groningen, University of Groningen, Groningen, The Netherlands; University College Groningen, University of Groningen, Groningen, The Netherlands; Department of Health Sciences, Unit Health Psychology, University Medical Centre Groningen, University of Groningen, Groningen, The Netherlands; Department of Gastroenterology and Hepatology, University Medical Centre Groningen, University of Groningen, Groningen, The Netherlands; The Henry D. Janowitz Division of Gastroenterology, Department of Medicine, Icahn School of Medicine at Mount Sinai, New York, NY, United States; Department of Gastroenterology and Hepatology, University Medical Centre Groningen, University of Groningen, Groningen, The Netherlands; Department of Health Sciences, Unit Health Psychology, University Medical Centre Groningen, University of Groningen, Groningen, The Netherlands

**Keywords:** Inflammatory bowel disease, fatigue, perceived stress, sleep problems, physical activity, longitudinal trajectories

## Abstract

**Background and Aims:**

Fatigue is a common and burdensome problem for patients with inflammatory bowel disease (IBD). Previous studies lack insight into the individual variability in fatigue severity and course over time, and the characteristics of patients at risk of severe and persistent fatigue. This study aimed to identify distinct groups of IBD patients based on their level and course of fatigue over 5 years. Subsequently, we examined the relationship between these trajectories, patient characteristics, and trajectories of perceived stress, sleep, and physical activity.

**Methods:**

This longitudinal cohort study used prospectively collected data from the myIBDcoach telemedicine monitoring tool, including 320 IBD patients who completed 3 or more online consultations between 2016 and 2021. Latent class growth analyses were performed to identify distinct trajectories.

**Results:**

We found 5 subgroups with distinct trajectories of fatigue, differing in level and course over time, with 33% of patients experiencing chronic stable and high levels of fatigue. Few differences in patient characteristics were found between trajectories, yet the chronic high fatigue subgroup was more likely to report persistent stable sleep problems, perceived stress, and little physical activity over time compared to the other groups.

**Conclusions:**

Distinct subgroups of IBD patients can be identified based on longitudinal fatigue trajectories. The relatively stable levels of fatigue, stress, sleep, and physical activity suggest that a one-time screening of patients on these topics may be sufficient to identify those at risk. Interventions aiming to reduce fatigue should target persistent stress, sleep problems, and low levels of physical activity.

## 1. Introduction

Inflammatory bowel diseases (IBDs) include Crohn’s disease (CD) and ulcerative colitis (UC), two chronic diseases characterized by inflammation of the gastrointestinal tract with a relapsing-remitting course of disease activity. One of the most frequently reported complaints by IBD patients is persistent fatigue.^1^ A recent meta-analysis estimated an overall prevalence of 47% for IBD-related fatigue (ranging from 24% to 87%),^2^ with generally higher prevalence rates in patients with active IBD (80%) compared to patients in remission (50%).^3^ Fatigue often has a detrimental impact on patients’ health-related quality of life and psychosocial functioning,^4^ including mood problems, difficulties in performing daily life activities, and difficulties in developing and maintaining a healthy lifestyle.^5,6^ This high and persistent burden often results in increased healthcare use and costs. In IBD patients, the presence of fatigue has been associated with a more than 1.5-fold increase in pharmacological and medical costs over a 12-month period.^7^ Moreover, fatigue has been identified as one of the most important determinants of work productivity loss in IBD patients, resulting in higher economic costs.^8^

The high prevalence, burden, and costs of IBD-related fatigue warrant adequate screening and treatment. However, despite an increasing research interest in IBD-related fatigue over the last decade, current knowledge about the course and etiology, and thus about potential treatment pathways, is still limited.^2^ Longitudinal studies found that fatigue in IBD patients is often persistent and may worsen over time.^9–14^ However, the great variation around the mean suggests individual variability in the course of fatigue over time. This variability has not been thoroughly examined, and therefore, it remains unclear whether distinct subgroups of IBD patients exist, characterized by different levels and trajectories of fatigue over time. Additionally, it remains unclear whether a subgroup can be identified that particularly experiences persistent high levels of fatigue, and how many patients fall into this category of persistent fatigue. This knowledge is important for developing a more personalized approach to fatigue management in IBD, particularly for those at risk of persistent or worsening fatigue. Therefore, the first aim of the current study was to examine whether it is feasible and clinically relevant to distinguish subgroups of IBD patients who vary in the severity of fatigue over time.

Moreover, it remains unclear whether IBD patients at risk of developing severe and/or chronic fatigue have certain demographic and clinical characteristics. Several cross-sectional studies suggested a higher prevalence of IBD-related fatigue in females^6,15–17^ and younger patients.^6,17,18^ In line with this, Klusmann and colleagues^13^ found that younger patients and females were at greater risk to be classified into a persistent severe fatigue trajectory. In other studies, however, sex^14,19^ and age^19^ have not been shown to relate significantly to fatigue. Literature about the role of marital status in IBD-related fatigue is inconclusive, with some reporting no significant association and others reporting higher levels of fatigue in patients who are not in a relationship.^20,21^ Regarding clinical characteristics, patients with CD^6,14,19^ and a shorter disease duration^6^ seem at greater risk for fatigue. Moreover, a higher body mass index (BMI) is often associated with increased fatigue.^16,22^ Medication is another clinical factor that has often been associated with IBD-related fatigue.^23^ Specifically, immunomodulators can be related to anemia, which might contribute to fatigue. Less commonly, fatigue can also be a direct adverse effect of IBD-related medication (eg, for 6-mercaptopurine and some biologicals).^23^ Altogether, these studies suggest that demographic and clinical variables may account for different levels of fatigue at one point in time. However, they do not provide insight into the characteristics of patients at risk of developing a certain pattern of fatigue over time. Therefore, the second aim of this study was to examine the extent to which demographic and clinical variables are related to longitudinal fatigue trajectories.

In addition to demographic and clinical patient characteristics, the role of sleep quality, perceived stress, and physical activity might be crucial in the context of IBD-related fatigue, as these factors are often found to be related to fatigue and can be targeted by interventions (ie, modifiable risk factors).^3,23^ Poor sleep quality is common in IBD patients and can be explained by immune responses,^23,24^ nocturnal IBD-related symptoms or pain,^23^ as well as stress.^24,25^ Moreover, sleep problems occur both during active disease and remission, and are generally associated with higher levels of fatigue when measured cross-sectionally.^3,23,24,26,27^ Studies using a longitudinal design show that, on a group level, sleep disturbance can be a strong predictor of the development and worsening of fatigue over time.^9,10^ Furthermore, stress and fatigue have been shown to affect each other, in the general population as well as in people with a chronic disease, with various theories explaining the bidirectional association between stress and fatigue.^28^ Findings of a few cross-sectional and longitudinal studies in IBD patients suggest that higher levels of psychological distress are generally associated with higher levels of fatigue.^10,15,29^ Physical activity is another factor that has been related to IBD-related fatigue.^18,30,31^ In general, patients with IBD tend to have a lower level of physical activity than healthy people,^31^ and physical inactivity has been found to contribute to IBD-related fatigue, potentially through reduced muscle mass and pro-inflammatory processes.^3,32^ Although some of these associations may seem self-evident, these factors are not always taken into account when identifying perpetuating factors of IBD-related fatigue and considering treatment options.^33,34^ To provide more insight into the role of these modifiable risk factors in the course of IBD-related fatigue, the last aim of this study was to examine individual differences among IBD patients in terms of sleep quality, perceived stress, and physical activity over time, and how these relate to the experience of fatigue over time.

To summarize, in order to optimize the screening and treatment of IBD-related fatigue, a better understanding is needed of individual differences in the prevalence of fatigue over time, and its associations with patient characteristics as well as modifiable risk factors. The primary aim of this study was to examine individual differences in the course of fatigue over 5 years’ time, by identifying subgroups of IBD patients with different longitudinal trajectories of fatigue. Identifying these subgroups may provide insight into the course of fatigue at an individual level, and the number of people experiencing a certain pattern of fatigue. The second aim was to examine demographic and clinical differences between the identified subgroups with distinct fatigue trajectories. Finally, we aimed to identify subgroups of IBD patients with varying trajectories of sleep problems, perceived stress, and physical activity over time, and to explore whether these subgroups relate to subgroups with distinct fatigue trajectories. The latter may provide greater insight into the role of modifiable risk factors in the severity and course of fatigue over time, potentially identifying new avenues for optimizing fatigue management for distinct groups of IBD patients.

## 2. Materials and methods

### 2.1. Study design and procedure

This longitudinal cohort study used prospectively collected data from the myIBDcoach telemedicine monitoring tool in the University Medical Centre Groningen (UMCG). Details of myIBDcoach have been described elsewhere.^35^ Briefly, myIBDcoach offers patients a platform to provide data on their functioning and have regular e-health consultations to monitor their disease. Topics that are covered during these online consultations include disease activity, medication use, treatment adherence, lifestyle factors, work status, and psychosocial well-being.

All patients with CD, UC, or IBD-undefined (IBD-U) in the UMCG have received the option to monitor their disease via this e-health application in addition to regular physical consultations in the hospital. The frequency of the e-health consultations differed per patient and varied between once per 6 months to once per year. The date of entering myIBDcoach (ie, baseline) was different for each patient. For the present study, we included all patients within the UMCG who completed 3 or more consultations through myIBDCoach between October 2016 and October 2021. Prior to performing the study, we determined that a minimum of 300 patients was needed to perform the statistical analysis.^36^ Detailed baseline data from the closest hospital visit to inclusion in myIBDcoach (ie, sex, age, type and classification of the disease, and disease duration) were collected from electronic patient files. The study was approved by the Medical Ethical Research Committee of the Maastricht University Medical Centre, being applicable to the UMCG (no. 2021-2758). All patients gave written informed consent to use their monitoring data for research purposes.

### 2.2. Measures

#### 2.2.1. Baseline demographic and clinical characteristics

Baseline demographic and clinical characteristics included age, sex (male/female), employment (paid employment/unpaid employment/both paid and unpaid employment/unemployed), marital status (in a relationship/not in a relationship), type of IBD diagnosis (CD/UC/IBD-U), age at diagnosis, disease duration, BMI, smoking (yes/no), type of IBD-related medication and Montreal classification^37^ according to disease type: A (age at diagnosis), B (behavior), E (extent), L (location), and S (severity). Type of medication was classified into aminosalicylates, corticosteroids, immunomodulators, and biologicals. Self-reported disease activity at baseline was measured with the Monitor IBD At Home (MIAH) questionnaire.^38^ Depending on the type of IBD, the MIAH includes questions regarding stool frequency, urgency, rectal bleeding, mucus, fatigue, abdominal pain, and patient-reported disease activity. To indicate active disease, the following cutoff points are recommended: MIAH-CD > 3.6 and MIAH-UC > 3.5.^38^ For IBD-U, the cutoff of the MIAH-UC was used.

#### 2.2.2. Fatigue, perceived stress, sleep problems, and physical activity

During the 5-year study period, all 4 outcomes were assessed with one patient-reported item for each domain of interest (Table 1). Fatigue and perceived stress were measured with a visual analog scale (VAS) on which a patient could report their current level of symptoms, with higher scores reflecting higher levels. Sleep quality and perceived stress were examined with a 5-point Likert scale.

**Table 1:** Overview of questions used in the myIBDcoach telemedicine tool.

Domain	Question	Scale
Fatigue	Rate your fatigue level in the past 24 hours on a scale from 0 to 10, with 0 being “not fatigued” and 10 being “extremely fatigued,” what number would you give?	VAS [0–10]
Sleep problems	Fill in this sentence: In the past month, I … slept well. 0. Always or almost always, 1. Often, 2. Regularly, 3. Occasionally, 4. Seldom or never.	Likert [0–4]
Perceived stress	Rate your stress level today on a scale from 1 to 10, with 1 being “no stress” and 10 being “extreme stress,” what number would you give?	VAS [1–10]
Physical activity	Fill in this sentence: In the past month, I … engaged in sufficient physical exercise to keep up or improve my strength and fitness level. 0. Never or once in a while, 1. Occasionally, 2. Regularly, 3. Almost every day, 4. Every day.	Likert [0–4]

Abbreviation: VAS, visual analog scale. The original Dutch version of the questions can be found in Supplementary Table 1.

### 2.3. Statistical analyses

Baseline demographic and clinical characteristics were presented using means, standard deviations (SDs), medians, interquartile ranges (IQRs), and frequencies, as appropriate. Time, in years, was handled as a continuous variable. The number of days since the start of the study (October 18, 2016) to each consultation in myIBDcoach was divided by the total number of days in that year (ie, 365, except for the period between October 18, 2019, and October 17, 2020, as this was a leap year). To examine subgroups with distinct longitudinal trajectories of fatigue, perceived stress, sleep problems, and physical activity, latent class growth analysis (LCGA) was performed for each of the variables using R-studio package “lcmm.”

To find the most representative models, we compared models with 1 to 8 subgroups for each variable separately, using a combination of multiple fit indices and an interpretation of the subgroup parameters by plotting the group trajectories for their theoretical sensibility and distinctiveness.^39^ Each model was fitted 100 times with different start values to avoid local maxima with a maximum number of 10 iterations.^40^ The best-fitting classification model was based on information criteria, including the Akaike Information Criterion (AIC) and the Bayesian Information Criterion (BIC), with lower values of AIC and BIC suggesting better model fit.^41^ Because AIC and BIC often show a decreasing function, we also considered the elbow cutoff point by visualizing the values. Entropy was used to inspect latent subgroup separation. Higher values of entropy indicate better separation between subgroups,^42^ with an entropy of at least 0.6 being considered as appropriate.^43^ The minimum number of people in each subgroup was set at 5%.

To examine whether subgroups with distinct fatigue trajectories were related to demographic (ie, age, sex, marital status) and clinical variables (ie, type of IBD, disease duration, BMI, and medication use), we performed chi-square tests for categorical variables and analysis of variance for continuous variables. Nonparametrical Kruskal–Wallis tests were performed in case the continuous data were not normally distributed. Statistical significance was set at *p* < 0.05. Subsequently, we performed three univariate chi-square tests to examine whether subgroups with distinct fatigue trajectories were related to subgroups of sleep problems, perceived stress, and physical activity. Post hoc tests were performed to examine which pairs of the subgroups were different from one another, after Bonferroni correction.

## 3. Results

### 3.1. Patient characteristics

The current study included 320 IBD patients. Demographic and clinical characteristics at baseline are presented in Table 2. More than half of the patients (56%) were female and patients were on average 44 years old (SD = 13). More than half of the patients (55%) were diagnosed with CD, with a median disease duration of 14 years (IQR = 13). The vast majority of patients were in remission at baseline (74% for CD, 86% for UC, and 94% for IBD-U).

**Table 2:** Sociodemographic and clinical patient characteristics at baseline* (*n* = 320).

Variable	*n* (%)	Statistics
Sex, *n* (%)	320 (100)	
Female		180 (56)
Male		140 (44)
Age (years), mean (SD)	320 (100)	43.7 (13.3)
Employment, *n* (%)	292 (91)	
Paid employment		221 (76)
Unpaid employment		16 (5)
Both paid and unpaid employment		4 (1)
Unemployed		51 (18)
Relationship status, *n* (%)	320 (100)	
In a relationship		229 (72)
Not in a relationship		91 (28)
BMI (kg/m^2^), mean (SD)	320 (100)	25.5 (4.7)
Current smoking, *n* (%)	320 (100)	
Yes		46 (14)
No		274 (86)
Disease type, *n* (%)	320 (100)	
CD		176 (55)
UC		122 (38)
IBD-U		22 (7)
Clinical disease activity^†^, *n* (%)	247 (77)	
CD—in remission		107 (74)
CD—active disease		37 (26)
UC—in remission		73 (86)
UC—active disease		12 (14)
IBD-U^‡^—in remission		17 (94)
IBD-U^‡^—active disease		1 (6)
Disease duration (years), median (IQR)	320 (100)	14 (13.0)
Age at diagnosis in years, median (IQR)	320 (100)	25.5 (15.3)
Age at diagnosis^§^, *n* (%)	320 (100)	
A1: <17 years		43 (13)
A2: 17–40 years		221 (69)
A3: >40 years		56 (18)
Disease extent at diagnosis^§^ [UC], *n* (%)	320 (100)	
E1: proctitis		13 (11)
E2: left-sided colitis		40 (33)
E3: pancolitis		68 (56)
Disease location at diagnosis^§^ [CD], *n* (%)	320 (100)	
L1: ileal		56 (32)
L2: colonic		36 (20)
L3: ileocolonic		86 (49)
L4: Upper GI disease		23 (13)
Disease behavior at diagnosis^§^ [CD], *n* (%)	320 (100)	
B1: nonstricturing, nonpenetrating		88 (50)
B2: stricturing		40 (23)
B3: penetrating		32 (18)
Perianal disease		49 (28)
Medication use, *n* (%)	317 (99)	
None		98 (31)
Aminosalicylates		108 (34)
Immunomodulators		114 (36)
Biologicals		66 (21)
Corticosteroids		35 (11)

Abbreviations: BMI, body mass index; CD, Crohn’s disease; IBD-U, IBD-unclassified; IQR, interquartile range; UC, ulcerative colitis.

*Patients entered myIBDcoach at different moments in time.

^†^According to the Monitor IBD At Home (MIAH) questionnaire.

^‡^Based on MIAH-UC cutoff.

^§^According to the Montreal classification.

### 3.2. Trajectories of fatigue

Following LCGA, the AIC value was lowest for the model with 6 subgroups and BIC was lowest for the model with 5 subgroups (see Supplementary Figure 1). Since we considered models with the lowest AIC and BIC, and, based on AIC and BIC values, there was no clear elbow point after the model with 2 subgroups, we considered models with 2 to 5 subgroups for interpretation. For each model, the entropy was above the cutoff point of 0.60 (0.83, 0.71, 0.68, and 0.66, respectively), and all subgroups contained more than 5% of the sample.

**Figure 1:**
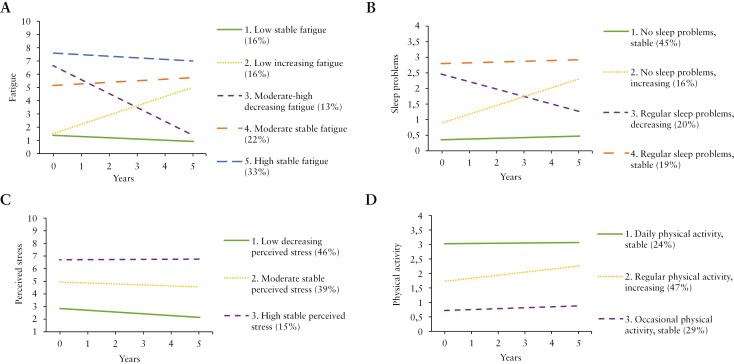
Subgroups with distinct trajectories of fatigue, sleep problems, perceived stress, and physical activity.

We identified 5 subgroups with distinct trajectories of fatigue (Figure 1a). Table 3 shows the parameter estimates of the subgroups. As can be observed, the 5 subgroups of patients differed in both level and course of fatigue over time, with subgroup 1 (low stable fatigue; 16%), 4 (moderate stable fatigue; 22%), and 5 (high stable fatigue; 33%) showing rather stable levels of fatigue over time. In contrast, patients in subgroup 2 (low increasing fatigue; 16%) and subgroup 3 (moderate–high decreasing fatigue; 13%) showed an increase and decrease in fatigue over time, respectively.

**Table 3:** Parameter estimates of trajectories of fatigue, sleep problems, perceived stress, and physical activity.

	Coefficient	SE	Subgroup size (%)
**Fatigue**			
1. Low stable fatigue			16
Intercept	1.41*	0.47	
Slope	−0.09	0.14	
2. Low increasing fatigue			16
Intercept	1.55*	0.57	
Slope	0.69*	0.22	
3. Moderate–high decreasing fatigue			13
Intercept	6.62*	1.19	
Slope	−1.04*	0.26	
4. Moderate stable fatigue			22
Intercept	5.17*	0.58	
Slope	0.11	0.16	
5. High stable fatigue			33
Intercept	7.59*	0.22	
Slope	−0.11	0.08	
Residual variance	1.83	0.04	
**Sleep problems**			
1. No sleep problems, stable over time			45
Intercept	0.36*	0.08	
Slope	0.02	0.03	
2. No sleep problems, increasing over time			16
Intercept	0.89*	0.20	
Slope	0.28*	0.08	
3. Regular sleep problems, decreasing over time			20
Intercept	2.45*	0.16	
Slope	−0.24*	0.05	
4. Regular sleep problems, stable over time			19
Intercept	2.80*	0.11	
Slope	0.02	0.04	
Residual variance	0.68	0.02	
**Perceived stress**			
1. Low decreasing perceived stress			46
Intercept	2.85*	0.21	
Slope	−0.14*	0.06	
2. Moderate stable perceived stress			39
Intercept	4.95*	0.26	
Slope	−0.08	0.09	
3. High stable perceived stress			15
Intercept	6.70*	0.40	
Slope	0.01	0.12	
Residual variance	1.70	0.04	
**Physical activity**			
1. Daily physical activity, stable over time			24
Intercept	3.03	0.13	
Slope	0.01	0.04	
2. Regular physical activity, increasing over time			47
Intercept	1.74*	0.09	
Slope	0.10*	0.03	
3. Occasional physical activity, stable over time			29
Intercept	0.72*	0.11	
Slope	0.03	0.04	
Residual variance	0.77	0.02	

*p < 0.05.

### 3.3. Differences in demographic or clinical variables between fatigue trajectories

The subgroups with distinct fatigue trajectories only differed significantly in sex (χ^2^ = 15.26, *p* < 0.01). Specifically, subgroup 5 (high stable fatigue; 33%) consisted of more females than males (68% vs 32%), whereas subgroup 1 (low stable fatigue; 16%) included more males than females (65% vs 35%). We found no significant differences between subgroups of fatigue trajectories in patients’ age (*F* = 2.18, *p* = 0.07), marital status (χ^2^ = 6.33, *p* = 0.61), BMI (*F* = 1.31, *p* = 0.27), type of IBD diagnosis (χ^2^ = 4.00, *p* = 0.41), and disease duration (*F* = 2.07, *p* = 0.08). Likewise, no significant differences were found based on medication use, such as aminosalicylates (χ^2^ = 3.33, *p* = 0.50), immunomodulators (χ^2^ = 6.92, *p* = 0.14), and biologicals (χ^2^ = 7.17, *p* = 0.13). For type of IBD and medication use, the expected counts for the categories IBD-U and corticosteroids were below 5. Therefore, these were excluded.

### 3.4. Trajectories of sleep problems, perceived stress, and physical activity

For sleep problems, the AIC value was lowest for the model with 5 subgroups and BIC was lowest for the model with 4 subgroups (Supplementary Figure 2). The elbow point appeared around the models with 2 or 3 subgroups. Therefore, models with 2 to 4 subgroups were considered. The entropy for these models was above the cutoff (0.84, 0.78, and 0.73, respectively), and each subgroup contained at least 5% of the sample. We identified 4 distinct trajectories for sleep problems (Figure 1b; Table 3). Patients in subgroups 1 (no sleep problems, stable; 45%) and 4 (regular sleep problems, stable; 19%) showed rather stable levels of sleep problems over time, whereas those in subgroups 2 (no sleep problems, increasing; 16%) and 3 (regular sleep problems, decreasing; 20%) indicated a change in sleep problems.

For perceived stress, the AIC value was lowest for the model with 5 subgroups and BIC was lowest for the model with 4 subgroups (Supplementary Figure 3). There was no clear elbow point after the model with 2 subgroups. Moreover, given a group size of less than 5% in the models with 4 or more subgroups, we only considered the models with 2 and 3 subgroups. The entropy for these models was above the cutoff of 0.60 (0.72 and 0.65, respectively). We identified 3 distinct trajectories for perceived stress (Figure 1c; Table 3). The 3 subgroups mainly differed in their level of perceived stress over time, with subgroup 1 (low decreasing perceived stress; 46%) indicating a low level of stress, subgroup 2 (moderate stable perceived stress; 39%) indicating a moderate level of stress, and subgroup 3 (high stable perceived stress; 15%) indicating a high level of perceived stress.

For physical activity, the AIC was lowest for the model with 4 subgroups, the BIC was lowest for the model with 3 subgroups, and the elbow point indicated the model with 3 subgroups (Supplementary Figure 4). Therefore, we considered the models with 2 and 3 subgroups for interpretation. For both models, entropy was above the cutoff (0.74 and 0.71, respectively), and subgroup size was at least 5%. We identified 3 distinct trajectories for physical activity (Figure 1d; Table 3). The 3 subgroups mainly differed in their level of physical activity over time, with subgroup 2 (regular physical activity, increasing; 47%) indicating an increasing level of physical activity over time, and subgroup 1 (daily physical activity, stable; 24%) and 3 (occasional physical activity, stable; 29%) showing a stable level of physical activity over time.

### 3.5. Associations between fatigue trajectories and trajectories of sleep problems, perceived stress, and physical activity

The subgroups with distinct fatigue trajectories were significantly related to subgroups of sleep problems (χ^2^ = 68.08, *p* < 0.05), perceived stress (χ^2^ = 50.45, *p* < 0.05), and physical activity (χ^2^ = 32.49, *p* < 0.05) (Table 4). Specifically, patients in subgroup 1 (low stable fatigue; 16%) were significantly more likely to report low levels of sleeping problems and stress over time, and regular to almost daily physical activity. In contrast, patients in subgroup 5 (high stable fatigue; 33%) were significantly more likely to report stable regular sleep problems and elevated stress over time, as well as only occasional physical activity. We found no significant differences in risk factors for fatigue in subgroup 2 (low increasing fatigue; 16%), 3 (moderate–high decreasing fatigue; 13%), and 4 (moderate stable fatigue; 22%).

**Table 4:** Number (%) of individuals in subgroups of fatigue, sleep problems, perceived stress, and physical activity.

	Fatigue				
	1. Low, stable	2. Low, increasing	3. Moderate–high, decreasing	4. Moderate, stable	5. High, stable
**Sleep problems**					
1. No, stable	43* (84%)	27 (53%)	22 (54%)	27 (39%)	24* (23%)
2. No, increasing	4 (8%)	12 (24%)	7 (17%)	7 (10%)	20 (19%)
3. Regular, decreasing	2* (4%)	8 (16%)	7 (17%)	19 (27%)	29 (27%)
4. Regular, stable	2* (4%)	4 (8%)	6 (14%)	17 (24%)	33* (31%)
**Perceived stress**					
1. Low, decreasing	39* (76%)	31 (61%)	20 (48%)	29 (41%)	29* (27%)
2. Moderate, stable	11 (22%)	15 (29%)	19 (45%)	32 (46%)	46 (43%)
3. High, stable	1 (2%)	5 (10%)	3 (7%)	9 (13%)	31* (29%)
**Physical activity**					
1. Daily, stable	22* (43%)	13 (26%)	12 (29%)	13 (19%)	16 (15%)
2. Regular, increasing	27 (53%)	22 (43%)	22 (52%)	35 (50%)	46 (43%)
3. Occasional, stable	2* (4%)	16 (31%)	8 (19%)	22 (31%)	44* (42%)

*p < 0.05 after Bonferroni correction, indicating a significantly higher/lower rate of individuals in this cell.

## 4. Discussion

With this longitudinal cohort study, we identified subgroups of IBD patients with distinct trajectories of fatigue over a period of 5 years. Moreover, we investigated differences in patient characteristics among these trajectories, and examined how fatigue trajectories relate to trajectories of perceived stress, sleep problems, and physical activity. We found 5 subgroups defined by distinct trajectories of fatigue, differing in level and course over time. Three trajectories were characterized by a rather stable level of fatigue over time, including one group of 33% of patients reporting persistently high levels of fatigue. The other 2 subgroups were defined by either an increase (16%) or a decrease (13%) in fatigue. Sex was the only demographic or clinical characteristic that was significantly related to trajectories of fatigue, with females being more likely than males to belong to the high persistent fatigue group. Moreover, we found that fatigue trajectories were related to trajectories of perceived stress, sleep problems, and physical activity. Patients in the subgroup reporting high stable fatigue levels were more likely to be classified in a subgroup experiencing regular sleep problems, high stress, and low physical activity.

One of the most meaningful findings is that one-third of the IBD patients reported high and persistent levels of fatigue for a substantial period of time (ie, years). This result adds to a growing body of longitudinal studies reporting a high prevalence and persistence of fatigue in IBD patients.^9–12^ By assessing fatigue beyond average levels and screening patients multiple times, our study enhances these findings by quantifying the proportion of patients with high and persistent fatigue. The identification of 5 distinct trajectories of IBD-related fatigue in our study supports the results of an earlier study by Klusmann and colleagues,^13^ which used a similar approach to examine subgroups of IBD patients with distinct fatigue trajectories. However, our study identified a significantly larger group of IBD patients with persistently high levels of fatigue, suggesting that more IBD patients might experience persistent fatigue than previously indicated. This difference is challenging to explain given the similarity in patient characteristics and approach (eg, statistical analyses). Perhaps the type of measurement influenced the results, as the outcomes in myIBDcoach were patient-reported, whereas those in the study of Klusmann and colleagues were reported by a doctor. Research shows that outcomes in IBD patients may differ depending on whether they are reported by a healthcare professional or the patient.^44^

Interestingly, we found few significant associations between subgroups of patients with distinct fatigue trajectories and their demographic or clinical characteristics, except that females were more likely than males to belong to the high persistent fatigue subgroup. Research conducted in both the general population and among individuals with a chronic disease consistently shows that severe and persistent fatigue is more prevalent in females compared to males.^6,13,15–17^ Biological factors (ie, hormone and stress-related processes), as well as the social context and living situation (eg, taking care of young children), seem to play an important role in this association.^45^ We found no other demographic characteristics related to fatigue trajectories. Previous studies were inconclusive regarding the role of age and marital status,^20,21^ with some suggesting that a younger age is associated with more severe fatigue,^6,13,17,18^ whereas others do not find this association.^46^ A higher prevalence of fatigue in younger patients may be related to a more active disease course, and social demands and challenges that might be less prominent later in life.^18,47^ One explanation for the nonsignificant result regarding age in our study might be that the majority of patients were of working age, resulting in a homogeneous group with comparable social roles and demands. The large number of patients (70%) who engaged in paid employment supports this idea. Considering that almost three-quarters of the patients in our cohort were in a relationship, a similar reasoning may be in place to explain why we did not find any differences in fatigue between patients who were in a relationship and those who were not.

Likewise, we found no significant associations between fatigue trajectories and clinical variables such as disease type, disease duration, and medication use. Although inconclusive, some previous studies suggest that these variables play a role in the experience of IBD-related fatigue.^6,14,19,23^ Our study included patients with a relatively long disease duration (median = 14 years), which might explain the lack of significant differences among the fatigue trajectories. It is plausible that patients with longer disease duration have developed more adaptive coping strategies compared to newly diagnosed patients, leading to a decrease in experienced fatigue.^6^ Additionally, over a longer disease course, medication may be adjusted appropriately, potentially reducing its impact on fatigue. Including more patients with a recent diagnosis might have revealed differences in disease duration and medication use among fatigue trajectories. Given that patients still experienced fatigue despite a long disease duration, the results indicate that factors unrelated to the disease and treatment may become more important as disease duration progresses, thereby supporting the biopsychosocial model of fatigue.^3^

We found consistent associations between fatigue trajectories and those of sleep, stress, and physical activity. A large number of patients were classified into subgroups reporting persistent regular sleep problems (19%), high perceived stress (15%), or low physical activity (29%). Patients in these risk groups were more likely to report high and persistent levels of fatigue. This result suggests that these modifiable risk factors play a role in the development and persistence of fatigue, which is in line with the current literature.^18,22,26^ Unfortunately, the low number of patients in certain subgroups refrained us from including all three risk factors into a prediction model (ie, multivariable logistic regression), and examining which of these factors is most important in the risk of developing a persistently high level of IBD-related fatigue. Future studies with larger sample sizes are required to examine the association between subgroups of IBD patients with distinct trajectories of fatigue and subgroups of sleep problems, perceived stress, and physical activity in a multivariable analysis, in order to determine the weight of the variables and control for other variables. Moreover, given the significant differences in fatigue trajectories between males and females, it would be interesting to examine in a larger study whether the associations with sleep, stress, and physical activity also differ by sex.

Another crucial aspect that requires more research and evidence is the causal direction of the associations between trajectories of perceived stress, sleep problems, physical activity, and IBD-related fatigue. Given that fatigue is assumed to be a multifactorial problem,^3^ it is likely that the associations between potential risk factors and IBD-related fatigue are complex and bidirectional. Other lifestyle or psychological factors might account for the associations (ie, confounders) or might interact with one another in their association with IBD-related fatigue (ie, moderators). For example, it is likely that the association between stress and fatigue is confounded by sleep disorders, physical inactivity, or disease-related functional limitations.^28^ Moreover, negative cognitions about fatigue and maladaptive coping strategies are also thought to contribute to the persistence of fatigue.^48^ Additionally, Davis and colleagues^18^ reasoned that physical activity may be a crucial mediating variable in the association between physiological factors of IBD (eg, disease activity), psychological factors (eg, distress), and fatigue. A recent qualitative study identified the role of vicious cycles in the experience and impact of pain and fatigue in IBD patients, indicating that multiple factors intensify and aggravate each other.^49^ In addition, questions may arise regarding the extent to which each of the current outcomes is modifiable, as we found increasing and decreasing trajectories for fatigue and sleep problems, but not for stress and physical activity. Since the current study is one of the first to examine these trajectories in IBD patients, it is difficult to draw conclusions about this. Studies using an intensive longitudinal design (eg, ecological momentary assessments) may shed light on the fluctuations of the outcomes over time, and the directions of the associations between the outcomes, on an individual level. For example, one study has reported that poor sleep appears to drive IBD-related fatigue on a day-to-day basis.^50^

### 4.1. Strengths and limitations

A strength of this study is the systematic and continuous monitoring of fatigue and potential risk factors over a time period of five years. The patient-centered and advanced statistical analyses (ie, LCGA) allowed us to explore individual differences in these variables and their associations over time. Nonetheless, some limitations need to be considered when interpreting our results. First, all variables were assessed with a single item from the telemedicine tool. The questionnaires in myIBDcoach were primarily designed to screen for several relevant aspects potentially associated with patients’ well-being in a clinical practice setting, and in order to limit patient burden and loss of adherence, only one item per domain was used. Although one item might not fully capture the concept of the outcomes, studies show that single-item questionnaires can correlate well with comprehensive questionnaires (eg, perceived stress).^51^ Second, all outcomes, including physical activity and sleep quality, were measured via self-report. Our results provide a first indication of the role of prolonged suboptimal levels of physical activity and sleep in IBD-related fatigue, which should be replicated using more objective measures. Third, we had no access to sufficient data on endoscopic scores or fecal calprotectin to indicate levels of disease activity throughout the study. Given that disease activity is found to play a role in fatigue, sleep problems, perceived stress, and physical activity, we recommend future studies to control for disease activity, preferably by the examination of endoscopic assessment or fecal calprotectin.^52^

### 4.2. Clinical implications

The current results suggest that 1 in 3 IBD patients is at risk of experiencing severe, persistent fatigue. Our results showed that these patients are more likely to report high levels of stress, sleep problems, and low physical activity. These findings contribute to the clinical understanding of IBD-related fatigue, by showing that, in addition to biomedical factors, cognitive and behavioral factors play a role in the development and course of fatigue.^3^ Monitoring sleep problems, stress, and low physical activity, in addition to regular clinical outcomes, may thus help to identify patients at risk of persistent fatigue. E-health tools such as myIBDcoach can facilitate this monitoring while minimizing resources needed.^53^ Moreover, as the individual patterns of sleep problems, stress, and physical activity remained fairly stable over time, it can be argued that a one-time screening can be used to identify patients at risk. Once at-risk patients are identified, the screening results can guide healthcare professionals in targeting which specific factors to treat in order to alleviate fatigue, hereby applying a biopsychosocial model of care.^22,23,54^ Although the temporality and causality between fatigue and the current modifiable risk factors require further investigation, our findings suggest that interventions aimed to improve sleep problems, stress, and physical activity might positively affect IBD-related fatigue. To date, an increasing number of studies showed that psychological interventions, particularly for stress and sleep problems, have beneficial effects in IBD patients,^55^ hereby replicating findings in other patient groups.^56^ Cognitive-behavioral therapy may be especially useful in the treatment of fatigue, as it addresses cognitive and behavioral factors that perpetuate fatigue, tailored to the patients’ needs.^57^ While reviews report mixed results regarding the effectiveness of structured physical activity interventions for reducing IBD-related fatigue,^58,59^ some evidence suggests that physical activity can also be a promising target for interventions offered to IBD patients, as it may not only improve fatigue and physical fitness but may also alleviate sleep and mood problems.^18^

## 5. Conclusion

This study shows the existence of distinct subgroups of IBD patients based on longitudinal trajectories of fatigue, sleep problems, perceived stress, and physical activity. Moreover, we found that female IBD patients, and patients with persistent high levels of sleep problems, stress, and low levels of physical activity, were more likely to experience chronic high levels of fatigue. This study underlines the importance of monitoring sleep problems, perceived stress, and physical activity in the context of IBD-related fatigue. Moreover, the findings suggest that management of these risk factors may be beneficial for reducing IBD-related fatigue.

## Supplementary Data

Supplementary data are available at *ECCO-JCC* online.

jjae163_suppl_Supplementary_Material

## Data Availability

The data and syntaxes that support the research findings are available upon reasonable request to the corresponding author. Data are not publicly available due to privacy and ethical restrictions.
