# Supplementary material for: The Substantial Role of Sleep, Stress, and Physical Activity in Persistent High Levels of Fatigue in Patients With Inflammatory Bowel Disease: A Longitudinal Trajectory Study
Source: J Crohns Colitis. 2024 Oct 26;19(4):jjae163. doi: 10.1093/ecco-jcc/jjae163 (PMC12001343; doi:10.1093/ecco-jcc/jjae163)
Supplement: jjae163_suppl_Supplementary_Material [file jjae163_suppl_supplementary_material.docx]

**Supplementary Materials**

The Substantial Role of Sleep, Stress, and Physical Activity in Persistent High Levels of Fatigue in Patients with Inflammatory Bowel Disease: A Longitudinal Trajectory Study

| **Supplementary Table 1.** Dutch translation of questions used in the myIBDcoach telemedicine tool | | |
| --- | --- | --- |
| Domain | Dutch question | Scale |
| Fatigue | Hoeveel last had u de afgelopen 24 uur van vermoeidheid, op een schaal van 0 tot 10? Op deze schaal betekent 0 'niet vermoeid', 10 betekent 'zeer vermoeid’. | VAS [0-10] |
| Sleep problems | Vul de volgende zin aan: In de afgelopen maand heb ik ...... goed geslapen.  0. Altijd of bijna altijd, 1. Vaak, 2. Regelmatig, 3. Af en toe, 4. Zelden of nooit. | Likert [0-4] |
| Perceived stress | Als u een waarde moet geven aan uw stress op een schaal van 1 tot 10, waar 1 geen stress betekent en 10 heel veel stress, hoe zou u vandaag uw stress omschrijven? | VAS [1-10] |
| Physical activity | Vul de volgende zin aan: In de afgelopen maand heb ik ...... gesport of voldoende lichaamsbeweging gehad om mijn kracht en conditie op peil te houden of te verbeteren.  0. Niet of een enkele keer, 1. Af en toe, 2. Met enige regelmaat, 3. Bijna elke dag, 4. Elke dag. | Likert [0-4] |
| *Note.* VAS = Visual Analogue Scale. | | |

| **Supplementary Figure 1.** AIC and BIC values per subgroup model of fatigue |
| --- |
|  |
| **Supplementary Figure 2.** AIC and BIC scores per subgroup model of sleep problems |
|  |
| **Supplementary Figure 3.** AIC and BIC scores per subgroup model of perceived stress |
|  |
| **Supplementary Figure 4.** AIC and BIC scores per subgroup model of physical activity |
|  |
